# Novel Blood-Derived Extracellular Vesicle-Based Biomarkers in Alzheimer’s Disease Identified by Proximity Extension Assay

**DOI:** 10.3390/biomedicines8070199

**Published:** 2020-07-07

**Authors:** Jonas Ellegaard Nielsen, Kamilla Sofie Pedersen, Karsten Vestergård, Raluca Georgiana Maltesen, Gunna Christiansen, Søren Lundbye-Christensen, Torben Moos, Søren Risom Kristensen, Shona Pedersen

**Affiliations:** 1 Department of Clinical Medicine, Aalborg University, DK-9000 Aalborg, Denmark; solc@rn.dk (S.L.-C.); srk@rn.dk (S.R.K.); 2 Department of Clinical Biochemistry, Aalborg University Hospital, DK-9000 Aalborg, Denmark; 3 BioXpedia A/S, DK-8200 Aarhus, Denmark; info@bioxpedia.com; 4 Department of Neurology, Aalborg University Hospital, DK-9000 Aalborg, Denmark; k.vestergaard@rn.dk; 5 Department of Anaesthesia and Intensive Care, Aalborg University Hospital, DK-9000 Aalborg, Denmark; rm@rn.dk; 6 Department of Biomedicine, Aarhus University, DK-8000 Aarhus, Denmark; gunna@loke.dk; 7 Department of Health Science and Technology, Aalborg University, DK-9220 Aalborg, Denmark; tmoos@hst.aau.dk; 8 Unit of Clinical Biostatistics, Aalborg University Hospital, DK-9000 Aalborg, Denmark

**Keywords:** Alzheimer, proximity extension assay, plasma, extracellular vesicles, biomarkers, mild cognitive impairment, protein

## Abstract

Easily accessible biomarkers for Alzheimer’s dementia (AD) are lacking and established clinical markers are limited in applicability. Blood is a common biofluid for biomarker discoveries, and extracellular vesicles (EVs) may provide a matrix for exploring AD related biomarkers. Thus, we investigated proteins related to neurological and inflammatory processes in plasma and EVs. By proximity extension assay (PEA), 182 proteins were measured in plasma and EVs from patients with AD (*n* = 10), Mild Cognitive Impairment (MCI, *n* = 10), and healthy controls (*n* = 10). Plasma-derived EVs were enriched by 20,000× *g*, 1 h, 4 °C, and confirmed using nanoparticle tracking analysis (NTA), western blotting, and transmission electron microscopy with immunolabelling (IEM). Presence of CD9^+^ EVs was confirmed by western blotting and IEM. No group differences in particle concentration or size were detected by NTA. However, significant protein profiles were observed among subjects, particularly for EVs. Several proteins and their ratios could distinguish cognitively affected from healthy individuals. For plasma TGF-α│CCL20 (AUC = 0.96, 95% CI = 0.88–1.00, *p* = 0.001) and EVs CLEC1B│CCL11 (AUC = 0.95, 95% CI = 0.86–1.00, *p* = 0.001) showed diagnostic capabilities. Using PEA, we identified protein profiles capable of distinguishing healthy controls from AD patients. EVs provided additional biological information related to AD not observed in plasma alone.

## 1. Introduction

Alzheimer’s dementia (AD) is the most common form of dementia, making up 60 to 80% of all cases [1], with a global prevalence of over 50 million by 2019 and an expected increase to 152 million by 2050 [2]. Current diagnostic methods include positron emission tomography scans and changes of amyloid-β (Aβ) and tau in the cerebrospinal fluid (CSF). However, insufficient accessibility for general practitioners, invasiveness, and high cost limit the usefulness of these methods [3]. Therefore, a reliable blood-based biomarker could be desirable. Biological pathways, such as systemic or inflammatory reactions are also implicated in AD pathogenesis, and e.g., a higher ApoE expression causes a higher systemic inflammation level, in addition to an increased vascular Aβ deposition [4,5]. Breakdown of the blood–brain barrier (BBB) is thought to occur as an early event of the AD pathogenesis leading to presence of proteins associated with neurodegeneration which may be reflected in blood [6], and the same applies for markers of potential endothelial damage in AD [7]. However, the complex composition of blood, complicates the detection of low abundant proteins [8].

Extracellular vesicles (EVs) denote a heterogeneous group of particles with a double lipid-layer membrane, which are released from cells in an evolutionary conserved manner. The two major groups of EVs are exosomes (50–150 nm) and microvesicles (100–1000 nm). During biogenesis EVs are loaded with various proteins, lipids, and miRNAs, implying that their composition potentially mirrors the physiological state of their parental cell [9]. EVs are considered rich sources for disease biomarkers, which has related them to the term “liquid biopsies” [10]. Studies have also shown that these entities are released by neurons, astrocytes, oligodendrocytes, and other cell types of the central nervous system (CNS) [11,12], and that they are able to cross the BBB with subsequent appearance in the circulation [13]. EVs occurring in plasma have been associated with various phenomena related to AD pathology, including synaptic dysfunction [14], dysregulation of neuronal differentiation, proliferation, and survival [15], complement activation [16], and vascular complications [17,18]. EVs have also been implicated in the spreading [19,20] or clearing [21,22] of the accumulating neurotoxic proteins Aβ and tau.

Discovery-based studies require large-scale measurements of multiple proteins based on methods like mass spectrometry, immunological methods, or combinations thereof. A variety of the mentioned methods have previously been used in studies of AD for investigating markers of disease in both plasma [23,24] and EVs [13,16,25,26] although often with conflicting results. Mass spectrometry is biased towards abundant proteins, making detection of low abundant proteins a challenge [27]. A novel method, proximity extension assay (PEA), combines immunological detection with quantitative polymerase chain reaction (qPCR). By this combination, a substantial scalability, sensitivity, and specificity can be achieved [28], where relative quantification of multiple proteins is possible. In addition, whereas many methods may require a considerable amount of sample material, PEA only requires a diminutive sample volume of as little as 1 μL [29], to detect as many as 92 proteins simultaneously.

Therefore, the aim of this study was to investigate the levels of plasma and EV-derived proteins from patients with AD and Mild Cognitive Impairment (MCI) and to compare these with protein levels to that of healthy individuals. Our working hypothesis was that there are differences in the levels of proteins involved in neurological and inflammatory processes among healthy and disease individuals, and that these differences can be measured by the PEA technique.

Using PEA, both plasma and EVs contained a panel of protein ratios able to distinguish healthy controls from AD patients. Furthermore, PEA analysis of EVs allowed for the identification of statistically significant protein signatures, providing additional disease relevant information not found in plasma. 

## 2. Experimental Section

### 2.1. Study Participants

For this study, 10 participants were included in each of the three groups: AD, MCI, and healthy controls. Patients with AD or MCI were enrolled consecutively from the Department of Neurology at Aalborg University Hospital. Patients with AD were diagnosed with mild to moderate AD based on the International Classification of Diseases and Related Health Problems 10th Edition (ICD_10_) criteria [30] and National Institute of Neurological and Communicative Disorders and Stroke and the Alzheimer’s Disease and Related Disorders Association (NINCDS-ADRDA) criteria [31], while MCI patients were diagnosed according to the Petersen criteria [32].

Blood samples from age- and sex-related healthy subjects were collected as controls from the blood bank at Aalborg University Hospital. Blood donors above 65 years of age were required to answer a questionnaire prior to blood donation, regarding their physical and mental state, such as having experienced fatigue, chest pain, or memory impairment. This study was approved by the North Denmark Region Committee on Health Research Ethics (N-20150010) and conducted according to the Declaration of Helsinki. All study participants signed a written informed consent at the inclusion of the study.

### 2.2. Sample Collection

Patient samples were collected at the time of diagnosis and before initiation of treatment. Peripheral blood was collected from the median cubital vein using a 21-gauge needle (Vacuette, Greiner Bio-One, Austria) in 9 mL 0.105 M (3.2%) trisodium citrate tubes (BD Vacutainer^®^, UK) and in 10 mL clot activator tubes (BD Vacutainer^®^, UK). Platelet free plasma and serum was obtained by a double centrifugation of 2500× *g* for 15 minutes at room temperature [33]. Plasma and serum collection were stopped 1 cm above the buffy coat and pellet, respectively, after the first and second centrifugation. Plasma and serum were snap-frozen in liquid nitrogen and stored at −80 °C until analysis.

### 2.3. Biochemical Analysis

Alanine transaminase, albumin, carbamide, cholesterol, creatinine, C-reactive protein (CRP), glucose, high density lipoprotein cholesterol, lactate dehydrogenase, low density lipoprotein (LDL) cholesterol, and triglycerides were measured from serum samples at the Department of Clinical Biochemistry, Aalborg University Hospital, using the Cobas 8000 Modular Analyzer (Roche Applied Science, Penzberg, Germany). Haemoglobin was also measured for the enrolled study participants.

### 2.4. Extracellular Vesicle Enrichment and Preparation

EVs were enriched from 1 mL plasma using centrifugation twice at 20,000× *g* for 1 hour at 4 °C in a multifuge 3 S-R with a fixed angle rotor (#3332, Heraeus, Hanau Germany). EVs were washed in 1 mL 0.22 µm filtered phosphate buffered saline (PBS) between centrifugations, while the final EV pellet was resuspended in 10 µL PBS. In preparation for PEA, EVs were mixed 1:4 with M-PER™ Mammalian Protein Extraction Reagent (Thermo Scientific, Rockford, IL, USA) with Pierce™ Protease Inhibitor Mini Tablets, EDTA-free (Thermo Scientific, Rockford, IL, USA) and processed according to manufacturer’s protocol (Procedure for Lysis of Suspension) [34].

### 2.5. Characterization of Extracellular Vesicles

#### 2.5.1. Nanoparticle Tracking Analysis

Nanoparticle tracking analysis (NTA) was conducted to measure both the concentration and size of particles in EV pellets using a LM10-HS system equipped with a blue 405 nm laser (Malvern Instruments Ltd., Malvern, UK) coupled with a Luca-DL EMCCD camera (Andor Technology, Belfast, UK). System settings were validated using 0.1 µm silica beads (Polysciences, Hirchberg, Germany). Applied settings were camera level 11, threshold 3 with blur 9 × 9. EV pellets were diluted in filtered PBS to ensure an optimal particle per frame count recommended by the manufacturer. For each sample, five video recordings of 30 seconds were processed by the Nanosight NTA software version 3.0 (Malvern Instruments Ltd., Malvern, UK). 

#### 2.5.2. Western Blotting

Western blotting was performed to validate EV enrichment using the EV markers CD9 and programmed cell death 6-interacting protein (ALIX). EV pellets were pooled and mixed 4:1 with 2× Laemmli Sample Buffer (Bio-Rad Laboratories, Hercules, CA, USA), and boiled for 5 minutes at 100 °C. Proteins were separated in MiniProtean TGX 4–15% gels (Bio-Rad Laboratories, Hercules, CA, USA) and transferred at 100 V for 1 hour onto Amersham Hybond 0.2 µm PVDF blotting membranes (GE Healthcare, Little Chalfont, UK). The membranes were blocked for 1 hour with 5% (*w*/*v*) skim milk blocking buffer, and then incubated with primary monoclonal mouse anti-CD9 antibody (clone M-L13, BD Pharmingen, San Diego, CA, USA) and primary polyclonal rabbit anti- ALIX antibody (Merck Millipore, Burlington, MA, USA) diluted 1:500 and 1:1000 in 2.5% (*w*/*v*) skim milk blocking buffer, respectively. Secondary antibodies used were horseradish peroxidase conjugated polyclonal goat anti-mouse immunoglobulins/HRP (Dako, Glostrup, Denmark) and Amersham ECL donkey anti-rabbit IgG, HRP-linked F(ab’)_2_ fragment (GE Healthcare, Little Chalfont, UK) both diluted 1:30,000 in 2.5% (*w*/*v*) skim milk blocking buffer. As positive and negative controls, a platelet lysate and PBS were used, respectively. Protein detection was performed using enhanced chemiluminescence Lumi-Light Western Blotting Substrate (Roche Diagnostics, Indianapolis, IN, USA) and captured with the Pxi4 system and GeneSys software version 1.5.4.0 (Syngene, Cambridge, UK).

#### 2.5.3. Transmission Electron Microscopy with Immunogold Labelling

Transmission Electron Microscopy (TEM) was performed to detect and confirm vesicular CD9^+^ structures in the EV pellets. TEM was performed as previously described [35]. Briefly, five microliters of pooled EV pellets were mounted for 30 seconds on carbon-coated, glow discharged 400 mesh Ni grids (SPI Supplies, Chester, PA, USA), stained with one drop 1% (*w*/*v*) phosphotungstic acid (PTA) pH 7.0 (Ted Pella, Caspilor AB, Lindingö, Sweden), and then dry-blotted using filter papers. For phenotypical characterization of the vesicular structures, TEM with immunogold labelling (IEM) was performed. EV pellets were mounted for 30 seconds on carbon-coated, glow discharged 400 mesh Ni grids, with three times washing with PBS followed by blocking using 0.5% ovalbumin (Sigma-Aldrich, St. Louis, MO, USA) in PBS. The grids then incubated for 30 minutes at 37 °C with primary monoclonal mouse anti-CD9 antibody (clone M-L13, BD Pharmingen, San Diego, CA, USA) diluted 1:50 in 0.5% ovalbumin in PBS. Afterwards, grids were washed thrice in PBS and incubated with secondary antibody 10 nm gold-conjugated goat anti-mouse (British BioCell, Cardiff, UK) diluted 1:25 in 0.5% ovalbumin in PBS. The grids were washed thrice with PBS and then incubated on three droplets of 1% cold fish gelatine (Sigma-Aldrich, St. Louis, MO, USA), 10 minutes per drop. Lastly, the grids were again washed thrice with PBS, stained with one drop of 1% (*w*/*v*) PTA pH 7.0, and blotted dry with filter papers. Images were obtained using a JEM-1010 transmission electron microscope (JEOL, Tokyo, Japan) operated at 60 keV coupled with an electron sensitive CCD camera (KeenView, Olympus, Tokyo, Japan). For determination of size, a grid size replica (2,160 lines/mm) and ImageJ software version 1.51j8 (NIH, Bethesda, MD, USA) were used.

### 2.6. Proximity Extension Assay

To ensure equal sample loading for PEA, protein concentrations of EVs were determined using the Pierce™ bicinchoninic acid assay kit (Thermo Scientific, Rockford, IL, USA) according to manufacturer’s protocol (microplate procedure) [36]. EV pellets were diluted equally to 0.71 µg/µL, which were within the recommended concentration range for PEA of 0.5–1.0 µg/µL [37].

The Neurology and Inflammation panels (Olink^®^ Bioscience, Uppsala, Sweden) were used to analyse plasma and EVs for 182 neurological- and inflammatory-related proteins comprised by the manufacturer [28,29]. Briefly, antibody probe pairs labelled with oligonucleotides were incubated overnight with samples. Antibody probe pairs in close proximity to each other would then hybridize. DNA polymerase was added to the samples for DNA amplification and quantification through microfluidic real-time qPCR (96.96 Dynamic Array™ Integrated Fluidic Circuit, Fluidigm BioMark). Cycle threshold values (Ct-values) from the PCR reaction were presented as arbitrary units and used for relative quantification as Normalized Protein Expression (NPX) values in a log2-scale. Using NPX Manager (Olink^®^ Bioscience, Uppsala, Sweden), NPX values were calculated in three steps. The extension control was subtracted from sample Ct-values to correct for intra-assay variation (∆Ct_(analyte)_ = Ct_(analyte)_ − Ct_(Extension Control)_). The interplate control was subtracted to correct for inter-assay variation (∆∆Ct_(analyte)_ = ∆Ct_(analyte)_ − ∆Ct_(Interplate Control)_). The last step to calculate the NPX values requires normalization against a correction factor, which was calculated by the manufacturer during panel validation (NPX_(analyte)_ = Correction Factor − ∆∆Ct_(analyte)_). Thus, a high Ct-value equalled a low protein concentration, and a high NPX value corresponded to a high protein concentration.

### 2.7. Statistical Analysis

Data were presented as mean ± standard deviation (SD) or median with interquartile range (IQR). Data distribution and equal variances were assessed through Shapiro-Wilks test and Levene’s test, respectively. Proteins were filtered based on the percentage of NPX values above the lower limit of detection (LOD); proteins with ≥ 70% above LOD in at least one group were included in the analysis. Primary and secondary comparisons were made, comparing AD patients with controls or comparing all three groups, respectively. The first comparison was to elucidate clear distinctions in protein profiles between healthy and disease, while the second comparison was to investigate changes related to disease progression. Depending on the group comparisons, parametric tests (Student’s t-test and analysis of variance (ANOVA) with Tukey’s honestly significant difference (HSD) post hoc test) or the equivalent non-parametric tests (Mann-Whitney U test and Kruskal Wallis H test with Bonferroni correction) were used. A permutation-based false discovery rate (FDR) was applied to account for multiple comparisons. Significance was at < 0.05. Prior to fold-change calculations and correlation analysis, NPX values were transformed from a logarithmic scale to a linear scale. Correlations were performed amongst FDR significant proteins, CSF measurements, and CRP levels using Pearson’s rho. 

Venn diagrams were created using Biovenn [38]. Supervised partial least squared discriminant analysis (PLSDA) was performed to identify proteins related to sample grouping. Data was autoscaled and then a five-fold Venetian-Blinds cross validation was employed. Models were visualised using scores and loadings plots. The scores plots represent each individual sample. Loadings plots were used to identify the most significant proteins discriminating AD and controls. Receiver operating characteristic (ROC) curves and their area under the curve (AUC) were made for ratios of NPX values for proteins which showed high covariance by PLSDA and for single proteins with FDR significance. Optimal cut-off points for sensitivity and specificity were determined by Youden’s index [39].

IBM SPSS Statistics 26 (SPSS, Chicago, IL, USA), MATLAB (R2017b, MathWorks, Natick, MA, USA), Perseus version 1.6.7.0 [40], and GraphPad Prism 7.01 (GraphPad Software, La Jolla, CA, USA) were used.

## 3. Results

### 3.1. Characteristics of Study Participants

Two patient groups with AD or MCI and a control group were included in this study. Characteristics of study participants, biochemical parameters, and clinical parameters were summarized in Table 1. All biochemical parameters were within the range of the reference intervals for all three groups, however, few subjects had elevated levels of triglycerides and LDL cholesterol (Table 1). Generally, cognitive test scores and measured CSF protein levels indicated that AD patients were at the mild stage (Table 1).

### 3.2. Characterisation of Extracellular Vesicles Enrichment

**Figure 1:**
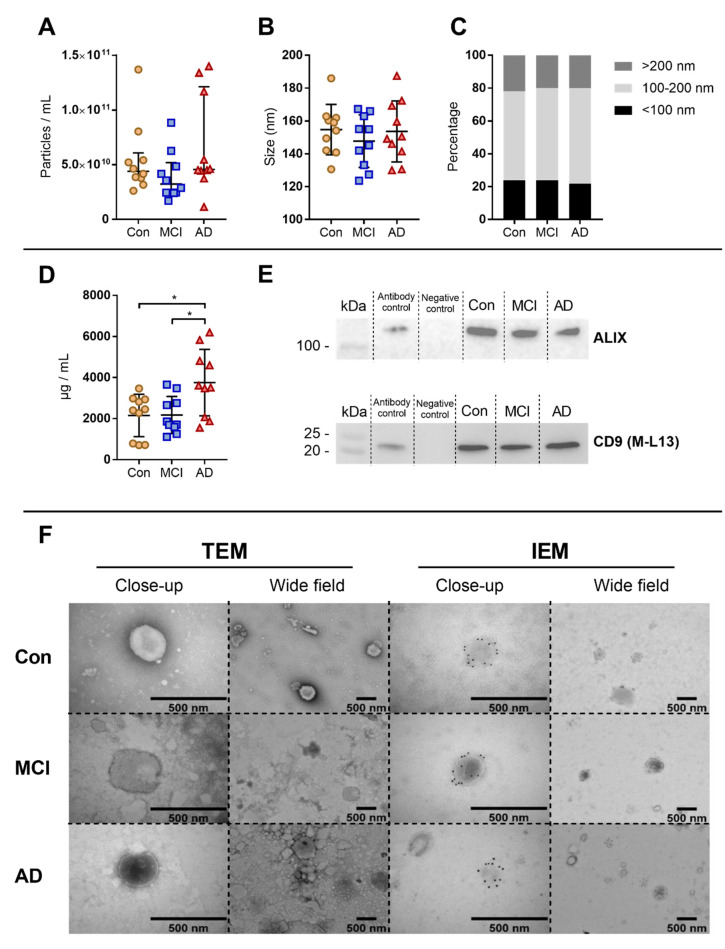
Extracellular vesicles characterization. Nanoparticle tracking analysis (NTA) was performed for controls and patient groups to determine (**A**) particle concentration and (**B**) mean particle size. (**C**) Size distributions of the measured particles were divided into three subgroups consisting of particles <100 nm, 100–200 nm, and >200 nm. (**D**) Protein concentrations of extracellular vesicles (EV) pellets measured by BCA. Alzheimer’s dementia (AD) patients had a significantly higher mean protein concentration compared to the Mild Cognitive Impairment (MCI) and controls groups. Scatter plots depict the median with interquartile range (IQR) and mean ± SD, respectively. (**E**) EV pellets were pooled within their respective groups and an equal volume of pellets were analysed by western blotting for the EV markers CD9 and ALIX. A positive signal for all markers were found in all three groups. (**F**) Transmission Electron Microscopy (TEM) images of negative stains of pooled EV pellets, as well as immunolabelling (IEM) images of CD9 positive particles identified in EV pellets. The immunoreactivity in the IEM images is mainly confined to the membrane of the EVs. Both close-up and wide field images are shown. The scale bar is 500 nm. * indicates *p* < 0.05.

NTA revealed that the particle concentration (Figure 1A), mean particle size (Figure 1B) and size distribution (Figure 1C) were not significantly different between healthy controls and patient groups. However, protein measurements showed a significantly increased protein concentration in AD patients compared to both MCI patients (*p* = 0.020) and healthy controls (*p* = 0.019) (Figure 1D). Western blot analysis showed the presence of the EV specific tetraspanin CD9 and the cytosolic protein ALIX in all three groups (Figure 1E), confirming the enrichment of EVs. TEM and IEM confirmed the presence of intact vesicular structures, as well as phenotypical confirmation of CD9^+^ EVs, within the size range of approximately 200 nm in EV pellets from both controls and patient groups (Figure 1F).

### 3.3. Multiplex Detection of Plasma and Extracellular Vesicle Protein Profiles by the Proximity Extension Assay

**Figure 2:**
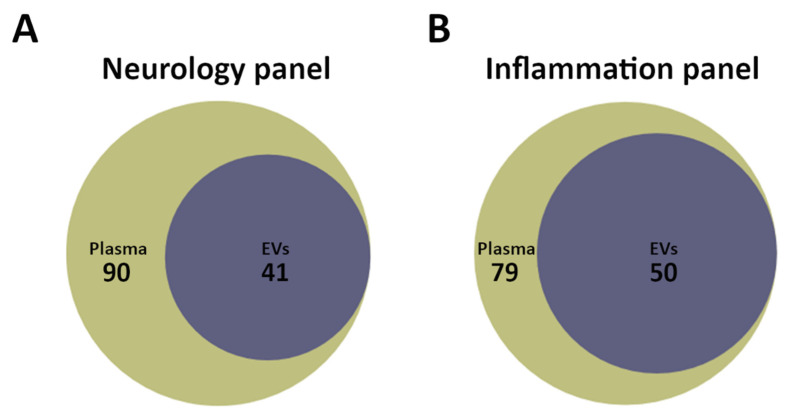
Area proportional Venn diagrams were created for visual representation of number of proteins in plasma and EV samples measured by proximity extension assay (PEA) using the Neurology and Inflammation panels. (**A**) For the Neurology panel, 90 proteins were detected in plasma with 41 proteins expressed in EV samples and 49 proteins only in plasma. (**B**) For the Inflammation panel, 79 proteins were detected in plasma with 50 proteins expressed in the EV samples and 29 proteins only in plasma.

The plasma and EV samples were analysed with the commercially available Olink^®^ Neurology and Inflammation PEA panels. The Neurology panel revealed a total of 90 proteins detected in plasma in all three groups, of which 41 was shared with the EVs (Figure 2A). For the Inflammation panel, 79 proteins were measured in plasma samples in all three groups, of which 50 proteins overlapped with the EVs (Figure 2B). None of the identified proteins, for both the panels, were uniquely expressed in the EV samples. A full list of all identified proteins and their corresponding NPX values from both panels can be found in supplementary materials Table S1.

**Figure 3:**
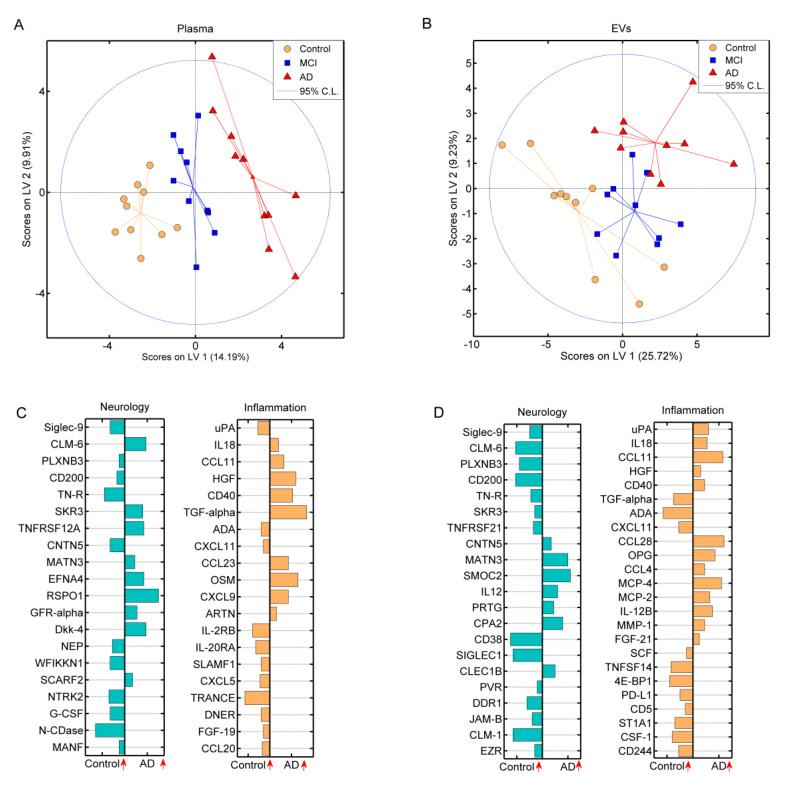
Partial least squared discriminant analysis of proteins from the Neurology and Inflammation panels measured in plasma and EV samples. Scores plots for all three groups measured in (**A**) plasma and (**B**) EV samples, where each score represents a sample. Loadings plots of (**C**) plasma and (**D**) EV samples, where each loading represents the variation in a specific protein. The larger the bar, the more significant the protein is in sample grouping. The orientation of each loadings’ variable describes if the corresponding protein was found at higher levels in controls compared to AD patients, where the vertical arrows were provided to facilitate interpretation.

PLSDA performed on plasma (Figure 3A) and EV (Figure 3B) derived proteins revealed samples clustering along the first latent variable (LV1) according to whether subjects were cognitively affected (AD or MCI) or not (control). A clear separation was observed between AD patients and controls in both scores plots, indicating that distinct protein profiles were causing this differentiation. Loadings plots revealed the protein profiles able to distinguish between AD and control individuals in both plasma (Figure 3C) and EV samples (Figure 3D). Thus, the sum of all loadings determined the averaged molecular signature of individuals.

### 3.4. Differentially Expressed Proteins Related to Alzheimer’s Dementia

In order to identify dysregulated proteins related to AD pathogenesis, primary and secondary comparisons were performed. Although MCI is a potential precursor stage for AD, it is also a heterogeneous group and as this patient group was included at time of diagnosis, there is an uncertainty to the outcome of their progression. Therefore, this group was excluded from the primary comparison to enable clear protein distinctions between AD and control individuals. A secondary comparison was subsequently conducted between all groups to investigate changes of protein expressions in regard to disease progression (Table 2 and Table 3). Additional information regarding post hoc test results from the secondary comparisons can be found in supplementary materials Table S2.

Two distinctive differentially expressed protein profiles were identified measuring the neurology related proteins comparing plasma and EV samples (Table 2). A higher number of significantly regulated proteins could be detected in EV samples compared to plasma, with 13 proteins detected from EVs and five proteins detected in plasma. Four proteins were significantly different between the three groups after FDR correction; CD38 (FDR: 0.022), CMRF35-like molecule 1 (CLM-1, FDR: 0.024), CMRF35-like molecule 6 (CLM-6, FDR: 0.027), and sialic acid-binding Ig-like lectin 9 (Siglec-9, FDR: 0.016), which were only differentially expressed in EV samples. For all four proteins, these were downregulated in the disease groups (MCI and AD) compared to healthy individuals.

Similar observations were noticed for the proteins measured by the Inflammation panel when comparing plasma and EVs (Table 3). As for the Neurology panel, the EV samples contained a higher number of significantly different proteins compared to plasma for the Inflammation panel. For plasma samples, eight significant proteins were identified, while for the EV samples 12 proteins were identified. Two proteins were significantly different after FDR correction when comparing AD patients and healthy controls; transforming growth factor-α (TGF-α, FDR: 0.020) from plasma samples and Eotaxin (CCL11, FDR: 0.024) from EV samples. Here both proteins were elevated in the AD group. This indicated that a great difference in protein expression could be obtained depending on the sample material analysed.

Several of the FDR significant proteins correlated amongst each other within the three groups. For AD patients Siglec-9 │ CLM-1 (rho = 0.78, *p* = 0.008), Siglec-9 │ CD38 (rho = 0.71, *p* = 0.02), CLM-1 │ CD38 (rho = 0.73, *p* = 0.02), and CLM-6 │ CD38 (rho = 0.77, *p* = 0.009) showed significant correlations. For the MCI group CLM-1 │ CLM-6 (rho = 0.91, *p* = 0.0002) showed significant correlation. Lastly, for the healthy control group Siglec-9 │ CLM-1 (rho = 0.72, *p* = 0.02), Siglec-9 │ CLM-6 (rho = 0.91, *p* = 0.0003), Siglec-9 │ CD38 (rho = 0.87, *p* = 0.001), CLM-1 │ CLM-6 (rho = 0.88, *p* = 0.001), CLM-1 │ CD38 (rho = 0.69, *p* = 0.03), and CLM-6 │ CD38 (rho = 0.91, *p* = 0.0003) showed significant correlations. Due to the immune related functions of the FDR significant proteins from the Neurology panel, correlations were also performed with CRP levels. However, no significant correlations were found. Inflammatory related proteins CCL11 and TGF-α did not show correlations with neither FDR significant proteins nor CRP. No significant correlations between CSF measurements and FDR significant proteins could be observed for the AD group.

**Figure 4:**
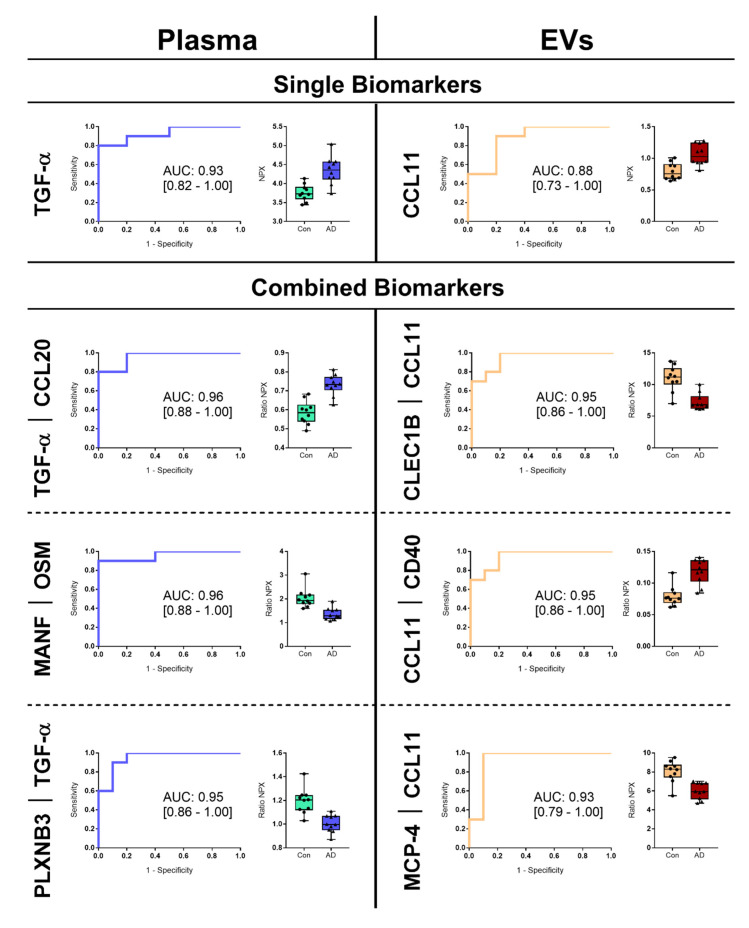
Receiver operating characteristic curves for single proteins with false discovery rate (FDR) correction, as well as top three protein ratios of proteins with high covariance found with partial least squared discriminant analysis (PLSDA), both for plasma and EVs. Area under the curves and 95% CI are presented for each receiver operating characteristic (ROC) curve, comparing healthy controls and AD patients. Box plots show the Normalized Protein Expression (NPX) and ratio NPX values for single or combined biomarker candidates comparing controls and AD patients.

ROC curves were made to ascertain diagnostic capabilities of proteins identified by the PLSDA models and FDR significance of single proteins for plasma and EVs. For both plasma and EV matrices, one protein and 19 protein ratios showed significance, with a set inclusion criteria for ROC curves with an AUC > 0.80 and with a 95% confidence interval (CI) 0.70–1.00 (Table 4). For single proteins with FDR significance; TGF-α (AUC: 0.93, 95% CI: 0.82–1.00, *p*: 0.001) for plasma and CCL11 (AUC: 0.86, 95% CI: 0.73–1.00, *p*: 0.004) for EVs showed noteworthy results for diagnostics when comparing controls and patients with AD (Figure 4). The top three combined biomarkers showed great diagnostic performance for plasma (ratios TGF-α and C-C motif chemokine 20 (CCL20) (AUC_TGF-α │ CCL20_: 0.96, 95% CI: 0.88–1.00, *p*: 0.001); mesencephalic astrocyte-derived neurotrophic factor (MANF) and oncostatin-M (OSM) (AUC_MANF │ OSM_: 0.96, 95% CI: 0.88–1.00, *p*: 0.001); plexin-B3 (PLXNB3) and TGF-α (AUC_PLXNB3 │ TGF-α_: 0.95, 95% CI: 0.86–1.00, *p*: 0.001)) and EVs (ratios c-type lectin domain family 1 member B (CLEC1B) and CCL11 (AUC_CLEC1B │ CCL11_: 0.95, 95% CI: 0.86–1.00, *p*: 0.001); CCL11 and CD40 (AUC_CCL11 │ CD40_: 0.95, 95% CI: 0.86–1.00, *p*: 0.001); monocyte chemotactic protein 4 (MCP-4) and CCL11 (AUC_MCP-4 │ CCL11_: 0.93, 95% CI: 0.79–1.00, *p*: 0.001)) when comparing healthy controls with AD patients (Figure 4).

Taken together, these results indicate that even though the majority of proteins detected were present in plasma, there is also important biological and potential diagnostic information to be investigated from EVs.

## 4. Discussion

In this study, we used PEA to examine protein profiles in plasma and EVs that could distinguish AD patients from healthy controls. Phenotypical and structural characterisation was performed and confirmed the enrichment of EVs. Several of the measured proteins were significantly different between patients and controls, and EV samples seemed to contain more differentially expressed proteins distinguishing the groups. 

NTA and TEM confirmed the presence of particles within the size range of EVs according to literature [41]. With respect to particle concentration and size, no significant differences were found between the three groups. Other studies have found increased levels of specific subpopulations of EVs in both CSF [41,42] and plasma [17,18] from AD patients, but NTA measures all particles and no specific subpopulations of EVs. AD patients presented with a significantly increased protein concentration in EV pellets compared to the other groups. Furthermore, EV enrichment was confirmed using the EV markers CD9 by western blot and IEM and ALIX by western blot.

Combining the Neurology and Inflammation panels for plasma and EVs separately, PLSDA determined a group of proteins in plasma and EVs, which could differentiate between the three groups. The PLSDAs provided a clear separation of the groups, with the EV samples containing the most significantly different proteins. Interestingly, when comparing controls with AD patients, five proteins; CLM-6, Contactin-5 (CNTN5), serine/threonine-protein kinase receptor R3 (SKR3), urokinase-type plasminogen activator (uPA), and TGF-α were expressed differently depending on sample material. This difference in expression could be due to the cargo selection mechanism during the biogenesis of EVs, which is currently not fully understood [10]. As mentioned, there is an uncertainty to the clinical outcome of the MCI patients, which could also be observed in the PLSDAs, as MCI samples clustered in-between the AD and control groups. This was especially clear for the EV samples, where few MCI patients overlapped with controls and AD patients.

Our results indicated that EVs contained more proteins that were significantly different compared to that from plasma. Similar observations were made by Gidlöf et al. [43], who compared plasma and EVs from patients with myocardial infarction to controls using PEA. EVs provided additional information that could not have been obtained with plasma alone [43]. In our study, four neurology related proteins were significantly different in EVs after FDR correction; CD38, CLM-1, CLM-6, and Siglec-9, with these proteins being less expressed in disease groups. The proteins showed statistically significant difference between MCI patients and controls, whereas the difference was not significant between AD patients and healthy controls, even though the proteins were expressed similarly in the AD group compared to the MCI group. This could be due to a larger variation in the AD group and the limited sample size. Mice deficient in CD38 had decreased levels of Aβ, and treatment of neuronal cell cultures with CD38 inhibitors lead to decreased Aβ secretion [44]. As we found decreased levels of CD38 in EVs for the cognitively affected, this could indicate a protective response. CLM-1 is an activating receptor participating in regulation of microglia [45]. Absence of CLM-1 lead to increased pro-inflammatory cytokine and nitric oxide production, and demyelination [46]. CLM-6 is an activator for monocytes [47] and acts as an inhibitor for T cell proliferation and activation [48]. As AD progresses, this could lead to a decreased expression of proteins with a protective function, which would agree with our findings, as we found decreased levels of these proteins for MCI and AD patients. To the author’s knowledge, no studies have reported any relation of Siglec-9 with AD. The functional murine equivalent to the human Siglec-9 is Siglec-E [49]. Siglec-E is found expressed on microglia [49,50], providing a protective effect by preventing phagocytosis of neural debris by microglia, thus reducing release of pro-inflammatory cytokines [51]. If a similar protective function of Siglec-9 could be elucidated, it would be in agreement with our finding of a decreased expression of this protein in the disease groups. Furthermore, TGF-α and CCL11 from the Inflammation panel were significantly upregulated in plasma and EV samples, respectively, comparing AD patients with controls. A study investigating neuroinflammation found that the cytokine TGF-α produced by microglia inhibits the pathogenic activities of astrocytes and that expression of TGF-α correlated with severity in multiple sclerosis lesions [52]. The chemokine CCL11 has been associated with ageing and neurodegeneration [53] and proposed as a risk factor for AD [54]. In agreement with the findings in our study, increased levels of CCL11 has been reported in CSF in vivo [54]. Testing the diagnostic capabilities of the combined protein ratios from the PLSDA models and the FDR significant proteins proved that both sample matrices contained important information regarding biomarker candidates for comparison of healthy controls and patients with AD. Interestingly, for plasma and EVs, TGF-α and CCL11, respectively, were involved in most of the combined biomarker models. These proteins were also the only FDR corrected proteins found significant with the mentioned inclusion criteria for ROC curves.

The peripheral immune system has been shown to be implicated in AD pathology, with infiltration of immune cells, e.g., macrophages to aid microglia with phagocytosis of Aβ [55], and cells of the immune system release EVs into the circulation [56]. Several of the FDR significant proteins have also been associated with immune cells, some of them such as Siglec-9 with neutrophils and monocytes [57], CLM-6 with monocytes and T cells [47,48], and CLM-1 and CCL11 with eosinophils [53,58]. This means that the observed expression of proteins in the current study could be due to a regulation of the peripheral immune system, as there is a cross talk between this and the CNS.

As indicated in the literature, these FDR significant proteins are involved in immunological processes and correlations showed that most of the expressions of these proteins were positively associated to each other, possibly indicating a functional association or response in regards to disease pathology. However, none of these proteins correlated with measured CRP levels. Neither, the two inflammatory proteins CCL11 and TGF-α correlated with CRP levels. This may indicate that their functions are not related to a systemic immunological reaction.

Whelan and colleagues have previously examined plasma from AD and MCI patients compared to controls using PEA [59]. They examined plasma and CSF also using the Neurology and Inflammation panels. Similar findings were observed, including changes in the protein’s junction adhesion molecule B (JAM-B), uPA, CD200, and CD38. JAM-B was significantly different in plasma and CSF comparing AD patients and healthy controls, and significantly different in plasma comparing MCI patients and healthy controls. We did not detect any differences in plasma; however, EVs depicted a similar difference of JAM-B, as that seen in the study by Whelan et al. [59], where JAM-B was found downregulated in cognitively affected. JAM-B is a tight junction protein expressed by brain endothelial cells forming the BBB [60], and its dysregulation could be ascribed to the disruption of the BBB [61]. JAM-B has also been associated with lymphocyte transendothelial migration [62] and vascular inflammation [63]. Similar observations could be observed for uPA, CD200, and CD38, hence strengthening our observations on differences between the three groups with downregulation of these proteins in disease groups. uPA has been shown to be important for recovery of axons after injury [64] together with ezrin (EZR) [65] which also was downregulated in our study, as well as inhibition of Aβ neurotoxicity [66]. The expression of uPA has been shown to be increased in cells stimulated by Aβ which through binding to uPA receptor (uPAR) and activation of plasminogen to plasmin can degrade Aβ [66,67]. The lower levels of uPA in plasma and EVs are not contradictory to this, but may indicate that uPA is bound to uPAR and plasminogen in the brain [68]. CD200 is thought to be involved in enhanced amyloid phagocytosis [69].

PEA has also previously been used to investigate EVs derived from neurons using the Neurology panel [70]. Proteins highly expressed in neuron-derived EVs were also of relevance in our study. These proteins included CLEC1B, CLM-6, epithelial discoidin domain-containing receptor 1 (DDR1), EZR, SKR3, tumour necrosis factor receptor superfamily member 21 (TNFRSF21), and PLXNB3 downregulated in AD, as well as interleukin 12 (IL12) and SPARC-related modular calcium-binding protein 2 (SMOC2) upregulated in AD. Another study reported of decreased levels of hepatocyte growth factor (HGF) in neuron-derived EVs from AD patients [15], which is in contrast to our findings showing increased levels of HGF in plasma from AD patients compared to controls. However, HGF have been shown to be increased in CSF of AD patients, possibly as a response to white matter damage [71], and it might be a similar response we observed in our plasma samples.

Our study heralds some limitations. At first, relatively low numbers of participants were included. However, we were able to observe a clear differentiation of AD patients from controls using the protein profiles from the PLSDA. Secondly, only larger EVs from the 20,000 × *g* enrichment was analysed by PEA, and therefore the information from smaller EVs have not been investigated. Thirdly, since PEA utilized panels of specifically selected proteins, other possibly relevant proteins were not measured. Fourthly, PEA only measured relative concentrations and no absolute levels of proteins making a direct comparison with other studies difficult. Due to this output of relative abundances, the PEA method is suitable as a screening device for discovery studies, however, absolute quantification using other methods would be needed for further validation studies. Fifthly, although all patients were clinically verified for MCI or AD, not all were examined for clinical markers such as CSF Aβ and tau, which could have been correlated to our proposed protein biomarkers. Sixthly, the controls were slightly younger than the patients were, since it was not possible to recruit older individuals, but this difference is probably only of minor importance.

Thus, the present results imply significant indications regarding the analysis of plasma- and EV- related proteins as putative biomarkers in AD, but our findings warrant further investigations using a larger independent cohort of cases and controls. It would also be of interest to include other types of dementia and more severe cases of AD to investigate if these proteins are specific to AD and if they follow disease progression.

## 5. Conclusions

Using PEA with selected protein panels related to neurological and inflammatory processes we identified protein profiles, using ratios of protein expressions based on the PLSDA models that could discriminate between healthy individuals and patients with AD. Comparison of protein panels between healthy controls and patient groups showed that EVs contained more significantly expressed proteins compared to plasma.

Thus, PEA was capable of identifying promising biomarker candidates for AD diagnostics for both plasma and EV samples. However, EVs contained additional information regarding disease pathology, which could not be obtained by plasma alone.

## Figures and Tables

**Table 1:** Characteristics of study participants.

	Subject Group			
	Con (*n* = 10)	MCI (*n* = 10)	AD (*n* = 10)	Reference Interval *
**Demographics**				
Age (years) ^b^	65 (65–66)	72 (69–76)	72 (68–74)	-
Male/female (*n*)	4/6	2/8	4/6	-
**Biochemical parameters**				
Alanine transaminase (U/I) ^b^	19.1 (18.0–34.7)	20.1 (15.4–45.4)	20.0 (18.0–29.5)	10.0–50.0
Albumin (g/L) ^a^	39.9 ± 2.3	39.4 ± 2.2	40.6 ± 2.5	34.0–45.0
Carbamide (mmol/L) ^b^	4.6 (4.3–6.1)	5.6 (5.2–7.1)	5.2 (4.8–6.4)	3.1–8.1
Cholesterol (mmol/L) ^a^	5.3 ± 0.7	5.8 ± 1.5	4.5 ± 0.9	4.1–8.5
Creatinine (µmol/L) ^b^	72.0 (60.8–86.1)	65.7 (60.5–80.1)	72.4 (66.9–88.9)	45.0–105.0
C-reactive protein (mg/L) ^b^	1.3 (0.5–3.2)	1.4 (0.6–3.7)	0.7 (0.3–2.6)	≤ 8.0
Glucose (mmol/L) ^b^	5.4 (4.8–5.6)	5.0 (4.5–5.6)	5.1 (4.9–5.4)	4.2–7.8
Haemoglobin (mmol/L) ^a^	9.0 ± 0.9	8.4 ± 0.8 (*n* = 8)	9.0 ± 0.5 (*n* = 9)	7.3–10.5
High density lipoprotein cholesterol (mmol/L) ^a^	1.5 ± 0.4	1.8 ± 0.5	1.7 ± 0.4	0.8–2.0
Lactate dehydrogenase (U/I) ^a^	164.2 ± 22.0	179.6 ± 32.6	181.4 ± 9.9	105.0–255.0
Low density lipoprotein cholesterol (mmol/L) ^a^	3.0 ± 0.7	3.3 ± 1.1	2.2 ± 0.6	1.8–4.5
Triglyceride (mmol/L) ^b^	1.5 (1.0–2.4)	1.3 (0.9–2.0)	1.1 (1.0–1.6)	≤ 2.0
**Clinical parameters**				
Mini Mental State Examination ^b^	-	27.5 (26.0–30.0)	25.5 (21.3–27.3)	-
Functional Activities Questionnaire ^a^	-	4.0 ± 2.0 (*n* = 3)	10.4 ± 4.6 (*n* = 5)	-
Addenbrooke’s Cognitive Examination^a^	-	85.0 ± 5.6 (*n* = 6)	58.7 ± 16.5 (*n* = 3)	-
CSF Aβ (ng/L) ^a^	-	998.5 ± 482.6 (*n* = 4)	626.3 ± 260.9 (*n* = 6)	> 500
CSF phospho-tau (ng/L) ^a^	-	98.0 ± 61.3 (*n* = 4)	80.5 ± 29.5 (*n* = 6)	< 61
CSF tau (ng/L) ^a^	-	563.0 ± 363.9 (*n* = 4)	628.2 ± 288.9 (*n* = 6)	< 450 (51–70 years) < 500 (71–90 years)

^a^ Mean ± SD. ^b^ Median with IQR. * Combined reference interval for men and women.

**Table 2:** Significantly expressed proteins identified from the Neurology panel in plasma and extracellular vesicles.

Neurology Panel—Plasma										
Protein	NPX			Fold Change			AD│Con		Con │MCI│AD	
	Con	MCI	AD	MCI│Con	AD│Con	AD│MCI	*p*-Value	FDR	*p*-Value	FDR
N-CDase	4.42 ± 0.58	4.49 ± 0.62	3.73 ± 0.39	1.07	0.60	0.56	0.006	0.212	0.006	0.436
RSPO1	3.83 ± 0.23	4.08 ± 0.24	4.20 ± 0.30	1.19	1.30	1.10	0.006	0.424	0.010	0.336
TNFRSF12A	6.00 ± 0.24	6.27 ± 0.28	6.27 ± 0.28	1.21	1.22	1.01	0.029	0.596	0.043	0.877
TN-R	4.24 ± 0.47	4.29 ± 0.47	3.81 ± 0.30	1.03	0.72	0.70	0.026	0.728	0.031	0.829
NTRK2	5.85 ± 0.24	5.96 ± 0.14	5.74 ± 0.17	1.07	0.92	0.86	0.247	1.000	<0.050	0.817
**Neurology Panel—EVs**										
CD38	0.94 ± 0.73	0.27 ± 0.20	0.24 ± 0.38	0.56	0.56	1.00	0.015	0.260	0.005	0.022
CLM-1	2.64 ± 0.72	1.70 ± 0.43	1.65 ± 0.64	0.49	0.50	1.00	0.004	0.136	0.001	0.024
CLM-6	2.33 ± 0.59	1.70 ± 0.23	1.75 ± 0.39	0.61	0.64	1.06	0.018	0.207	0.004	0.027
JAM-B	5.08 (4.52–5.42)	4.13 (3.87–4.64)	4.33 (3.99–4.87)	0.51	0.60	1.16	0.029 ^a^	0.194	0.029 ^b^	0.101
Siglec-9	3.76 ± 0.69	2.85 ± 0.31	3.06 ± 0.50	0.49	0.59	1.20	0.019	0.132	0.002	0.016
SIGLEC1	3.10 ± 1.29	2.29 ± 0.48	1.89 ± 0.70	0.41	0.33	0.81	0.019	0.164	0.017	0.089
SKR3	5.73 ± 1.05	4.78 ± 0.46	4.83 ± 0.75	0.43	0.47	1.09	0.041	0.218	0.020	0.067
CD200	1.12 ± 0.83	0.36 ± 0.38	0.65 ± 0.51	0.54	0.67	1.25	0.148	0.245	0.030	0.080
CLEC1B	8.52 ± 1.16	7.78 ± 0.62	7.50 ± 0.97	0.51	0.48	0.95	0.048	0.225	0.062	0.143
EZR	4.30 (3.24–4.63)	3.20 (2.30–3.70)	3.66 (3.37–4.10)	0.48	0.64	1.34	0.350	0.435	0.021^b^	0.075
gal-8	5.06 ± 0.87	4.17 ± 0.43	4.49 ± 0.73	0.48	0.65	1.37	0.131	0.229	0.020	0.067
PLXNB3	1.42 ± 0.52	0.91 ± 0.30	1.02 ± 0.33	0.68	0.73	1.08	0.052	0.212	0.019	0.081
TN-R	1.39 ± 0.47	1.03 ± 0.39	0.90 ± 0.47	0.77	0.71	0.93	0.032	0.199	0.056	0.137

^a^ Mann-Whitney U; ^b^ Kruskal Wallis H test with Bonferroni correction.

**Table 3:** Significantly expressed proteins identified from the Inflammation panel in plasma and extracellular vesicles.

Inflammation Panel—Plasma										
Protein	NPX			Fold Change			AD│Con		Con │MCI│AD	
	Con	MCI	AD	MCI│Con	AD│Con	AD│MCI	*p*-Value	FDR	*p*-Value	FDR
IL-2RB	1.29 ± 0.28	1.04 ± 0.18	1.03 ± 0.26	0.83	0.83	1.00	0.041	0.442	0.036	0.799
OSM	2.85 ± 0.56	3.39 ± 0.72	4.08 ± 1.08	1.52	2.84	1.87	0.005	0.150	0.009	0.338
TGF-α	3.76 ± 0.22	4.09 ± 0.42	4.34 ± 0.37	1.30	1.53	1.18	<0.000	0.020	0.003	0.208
TRANCE	5.09 ± 0.36	4.90 ± 0.43	4.49 ± 0.72	0.89	0.71	0.80	0.030	0.477	0.048	0.631
CXCL1	7.45 ± 0.75	7.05 ± 0.43	6.84 ± 0.45	0.70	0.61	0.87	0.039	0.499	0.061	0.703
CXCL9	7.63 ± 0.61	8.32 ± 1.09	8.36 ± 0.92	2.10	1.81	0.86	0.049	0.395	0.139	1.000
GDNF	2.35 ± 0.25	2.65 ± 0.34	2.38 ± 0.22	1.24	1.02	0.82	0.786	0.940	0.042	0.693
HGF	8.21 ± 0.35	8.50 ± 0.43	8.60 ± 0.39	1.24	1.32	1.06	0.029	0.607	0.081	0.717
**Inflammation Panel–EVs**										
4E-BP1	1.66 ± 0.65	1.11 ± 0.32	1.12 ± 0.42	0.64	0.65	1.02	0.040	0.345	0.025	0.189
ADA	1.81 ± 0.72	1.19 ± 0.26	1.24 ± 0.26	0.59	0.61	1.03	0.029	0.364	0.010	0.396
CCL11	0.75 (0.68–0.91)	0.78 (0.69–1.00)	1.03 (0.93–1.25)	1.02	1.21	1.19	0.001	0.024	0.011 ^b^	0.185
CD244	5.84 ± 0.96	4.96 ± 0.39	4.93 ± 0.69	0.47	0.49	1.05	0.026	0.435	0.012	0.224
CD40	10.15 ± 1.13	9.17 ± 0.50	9.00 ± 0.93	0.42	0.43	1.02	0.024	0.590	0.016	0.201
TGF-α	0.41 ± 0.26	0.14 ± 0.28	0.13 ± 0.28	0.83	0.83	1.00	0.034	0.338	0.049	0.149
CD5	5.62 ± 1.19	4.45 ± 0.68	4.88 ± 0.57	0.36	0.47	1.31	0.091	0.372	0.016	0.151
CXCL1	3.48 ± 1.03	3.03 ± 0.47	2.64 ± 0.71	0.63	0.53	0.84	0.048	0.307	0.069	0.196
IL-18R1	5.26 ± 1.04	4.38 ± 0.60	4.73 ± 0.43	0.49	0.59	1.22	0.155	0.386	0.041	0.176
SCF	1.89 (1.55–2.60)	1.94 (1.81–2.22)	1.48 (1.30–1.87)	1.03	0.75	0.73	0.071	0.328	0.034 ^b^	0.160
TNFRSF9	1.42 (1.02–1.69)	0.91 (0.77–1.16)	1.20 (0.98–1.48)	0.70	0.86	1.22	0.739 ^a^	0.945	0.033 ^b^	0.151
uPA	5.06 (4.60–5.53)	4.22 (3.95–4.61)	4.37 (4.21–4.54)	0.56	0.63	1.12	0.052 ^a^	0.347	0.042 ^b^	0.166

^a^ Mann-Whitney U; ^b^ Kruskal Wallis H test with Bonferroni correction.

**Table 4:** Receiver operating characteristic curves for protein ratios and single proteins for plasma and extracellular vesicles comparing healthy controls with AD patients.

Protein(s)	AUC	Sensitivity (%)	Specificity (%)	95% CI	*p*-Value
**Plasma**					
TGF-α	0.93	80	100	0.82–1.00	0.001
TGF-α │ CCL20	0.96	100	80	0.88–1.00	0.001
MANF │ OSM	0.96	90	100	0.88–1.00	0.001
PLXNB3 │ TGF-α	0.95	90	90	0.86–1.00	0.001
TGF-α │ FGF-19	0.95	80	100	0.86–1.00	0.001
OSM │ CCL20	0.94	80	90	0.84–1.00	0.001
GFR-α-1 │ TGF-α	0.94	100	80	0.84–1.00	0.001
TGF-α │ DNER	0.93	90	80	0.82–1.00	0.001
TN-R │ TGF-α	0.93	90	90	0.82–1.00	0.001
OSM│ CXCL5	0.93	90	90	0.82–1.00	0.001
PLXNB3 │ RSPO1	0.93	90	90	0.82–1.00	0.001
uPA │ TGF-α	0.92	90	80	0.80–1.00	0.001
TGF-α │ CD40	0.92	90	80	0.80–1.00	0.001
SKR3 │ TGF-α	0.91	70	100	0.79–1.00	0.002
TGF-α │ CCL23	0.91	80	90	0.79–1.00	0.002
N-CDase │ TGF-α	0.91	90	80	0.78–1.00	0.002
CNTN5 │ TGF-α	0.91	90	90	0.78–1.00	0.002
Siglec-9 │ RSPO1	0.89	90	80	0.73–1.00	0.003
Siglec-9 │ TGF-α	0.89	100	80	0.73–1.00	0.003
N-CDase │ HGF	0.88	80	90	0.73–1.00	0.004
**EVs**					
CCL11	0.88	90	80	0.73–1.00	0.004
CLEC1B │ CCL11	0.95	100	80	0.86–1.00	0.001
CCL11 │ CD40	0.95	100	80	0.86–1.00	0.001
MCP-4 │ CCL11	0.93	100	90	0.79–1.00	0.001
CD244 │ CCL11	0.92	100	80	0.79–1.00	0.001
SKR3 │ CCL11	0.92	100	90	0.77–1.00	0.001
JAM-B │ CCL11	0.92	100	90	0.77–1.00	0.001
SIGLEC1 │ CCL11	0.91	90	80	0.78–1.00	0.002
OPG │ CCL11	0.91	90	80	0.78–1.00	0.002
uPA │ CCL11	0.91	90	80	0.78–1.00	0.002
CCL11 │ MCP-2	0.91	100	70	0.78–1.00	0.002
CLM-1 │ CCL11	0.91	100	80	0.78–1.00	0.002
CCL11 │ CD5	0.91	90	90	0.76–1.00	0.002
Siglec-9 │ CCL11	0.90	90	80	0.76–1.00	0.002
CLM-6 │ CCL11	0.90	90	90	0.75–1.00	0.002
SMOC2 │ OPG	0.89	100	80	0.72–1.00	0.003
IL18 │ 4E-BP1	0.88	90	80	0.73–1.00	0.004
CCL11 │ PD-L1	0.88	80	90	0.73–1.00	0.004
PLXNB3 │ CCL11	0.88	90	80	0.72–1.00	0.004
CCL11 │ ADA	0.88	90	80	0.71–1.00	0.004

## Supplementary Materials

The following are available online at https://www.mdpi.com/2227-9059/8/7/199/s1, Table S1: Supplementary Material Table S1–Raw NPX values, Table S2: Supplementary Material Table S2—Post hoc p-values.
