# Supplementary material for: Novel Blood-Derived Extracellular Vesicle-Based Biomarkers in Alzheimer’s Disease Identified by Proximity Extension Assay
Source: Biomedicines. 2020 Jul 7;8(7):199. doi: 10.3390/biomedicines8070199 (PMC7400538; doi:10.3390/biomedicines8070199)
Supplement: Supplementary file 1 [file biomedicines-08-00199-s001.zip › Supplementary Material Table S2 - Post hoc p-values.docx]

**Table S2.** Post hoc *p*-values from the secondary comparisons.

| **Neurology** | | | | **Inflammation** | | | |
| --- | --- | --- | --- | --- | --- | --- | --- |
| **Plasma** | | | | | | | |
| **Protein** | **Post hoc *p*-value** | | | **Protein** | **Post hoc *p*-value** | | |
|  | Con│MCI | AD│Con | AD│MCI |  | Con│MCI | AD│Con | AD│MCI |
| N-CDase | 0.952 | **0.021** | **0.010** | IL-2RB | 0.071 | 0.056 | 0.994 |
| RSPO1 | 0.091 | **0.008** | 0.540 | OSM | 0.316 | **0.006** | 0.162 |
| TNFRSF12A | 0.078 | 0.067 | 0.997 | TGF-α | 0.095 | **0.002** | 0.250 |
| TN-R | 0.959 | 0.075 | **0.042** | TRANCE | 0.708 | **0.043** | 0.203 |
| NTRK2 | 0.426 | 0.393 | **0.039** | CXCL1 | 0.254 | 0.053 | 0.690 |
|  |  |  |  | CXCL9 | 0.211 | 0.178 | 0.995 |
|  |  |  |  | GDNF | 0.057 | 0.970 | 0.092 |
|  |  |  |  | HGF | 0.225 | 0.080 | 0.844 |
| **EVs** | | | | | | | |
| CD38 | **0.014** | **0.010** | 0.990 | 4E-BP1 | **0.044** | **0.048** | 0.999 |
| CLM-1 | **0.005** | **0.003** | 0.980 | ADA | **0.017** | **0.028** | 0.976 |
| CLM-6 | **0.008** | **0.014** | 0.964 | CCL11 | 1.000 | **0.013** | 0.071 |
| JAM-B | 0.033 | 0.151 | 1.000 | CD244 | **0.027** | **0.023** | 0.996 |
| Siglec-9 | **0.002** | **0.016** | 0.631 | CD40 | 0.052 | **0.021** | 0.911 |
| SIGLEC1 | 0.124 | **0.015** | 0.594 | TGF-α | 0.087 | 0.074 | 0.997 |
| SKR3 | **0.033** | **0.044** | 0.991 | CD5 | **0.013** | 0.146 | 0.510 |
| CD200 | **0.024** | 0.215 | 0.528 | CXCL1 | 0.399 | 0.056 | 0.514 |
| CLEC1B | 0.211 | 0.059 | 0.783 | IL-18R1 | **0.033** | 0.261 | 0.544 |
| EZR | **0.017** | 0.612 | 0.402 | SCF | 1.000 | 0.151 | **0.041** |
| gal-8 | **0.022** | 0.185 | 0.563 | TNFRSF9 | 0.051 | 1.000 | 0.105 |
| PLXNB3 | **0.020** | 0.074 | 0.828 | uPA | 0.051 | 0.180 | 1.000 |
| TN-R | 0.188 | 0.054 | 0.797 |  |  |  |  |
